# PBPK Modeling of Azithromycin Systemic Exposure in a Roux-en-Y Gastric Bypass Surgery Patient Population

**DOI:** 10.3390/pharmaceutics15112520

**Published:** 2023-10-24

**Authors:** Suvarchala Kiranmai Avvari, Jaclyn A. Cusumano, Vamshi Krishna Jogiraju, Pooja Manchandani, David R. Taft

**Affiliations:** 1 Samuel J. and Joan B. Williamson Institute for Pharmacometrics, Division of Pharmaceutical Sciences, Arnold & Marie Schwartz College of Pharmacy and Health Sciences, Long Island University, Brooklyn, NY 11201, USA; suvarchala.avvari@simulations-plus.com; 2 Division of Pharmacy Practice, Arnold & Marie Schwartz College of Pharmacy and Health Sciences, Long Island University, Brooklyn, NY 11201, USA; jaclyn.cusumano@liu.edu; 3 Gilead Sciences Inc., Foster City, CA 94404, USA; vamshi.jogiraju@gilead.com; 4 Astellas Pharma US, Northbrook, IL 60062, USA; pooja.manchandani@astellas.com

**Keywords:** azithromycin, Roux-en-Y, pharmacokinetics, PBPK modeling

## Abstract

In this investigation, PBPK modeling using the Simcyp^®^ Simulator was performed to evaluate whether Roux-en-Y gastric bypass (RYGB) surgery impacts the oral absorption and bioavailability of azithromycin. An RYGB surgery patient population was adapted from the published literature and verified using the same probe medications, atorvastatin and midazolam. Next, a PBPK model of azithromycin was constructed to simulate changes in systemic drug exposure after the administration of different oral formulations (tablet, suspension) to patients pre- and post-RYGB surgery using the developed and verified population model. Clinically observed changes in azithromycin systemic exposure post-surgery following oral administration (single-dose tablet formulation) were captured using PBPK modeling based on the comparison of model-predicted exposure metrics (C_max_, AUC) to published clinical data. Model simulations predicted a 30% reduction in steady-state AUC after surgery for three- and five-day multiple dose regimens of an azithromycin tablet formulation. The relative bioavailability of a suspension formulation was 1.5-fold higher than the tablet formulation after multiple dosing. The changes in systemic exposure observed after surgery were used to evaluate the clinical efficacy of azithromycin against two of the most common pathogens causing community acquired pneumonia based on the corresponding AUC_24_/MIC pharmacodynamic endpoint. The results suggest lower bioavailability of the tablet formulation post-surgery may impact clinical efficacy. Overall, the research demonstrates the potential of a PBPK modeling approach as a framework to optimize oral drug therapy in patients post-RYGB surgery.

## 1. Introduction

Obesity is a public health crisis, and it is estimated that 42.5% of U.S. adults aged 20 and older are obese and 9% are morbidly obese (BMI of >40 kg/m^2^ or >35 kg/m^2^ in the presence of comorbidities) [1]. The treatment of obesity is challenging, and bariatric surgery is the main treatment option to achieve a high rate of sustainable weight loss for morbid obesity [2]. Among the surgical options available, Roux-en-Y gastric bypass surgery (RYGB) has been considered the “gold-standard” method of bariatric surgery for decades [3].

**Figure 1:**
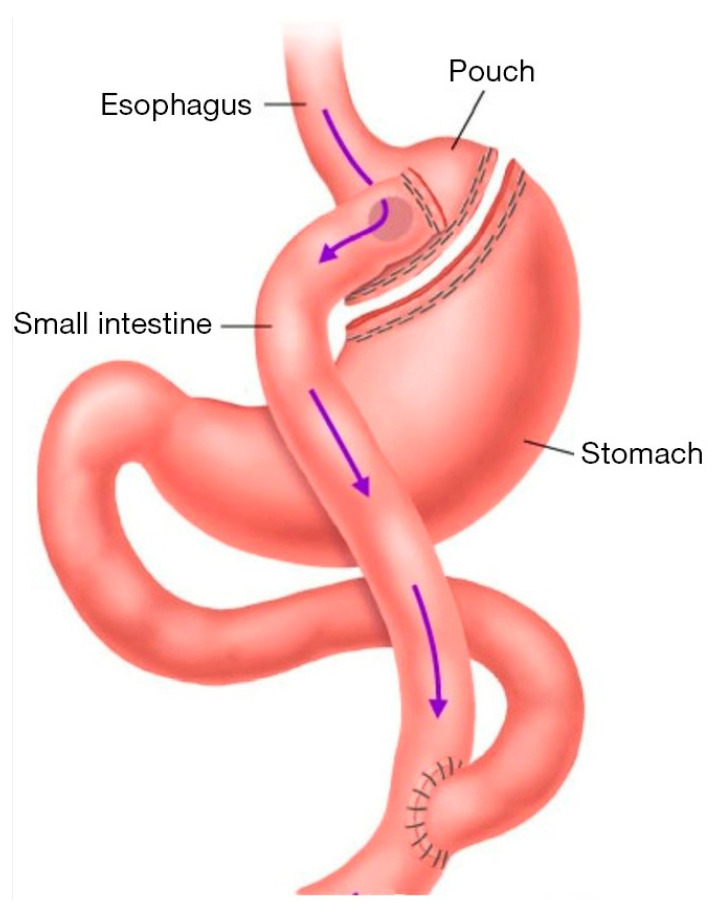
Effect of RYBG surgery on GI anatomy. The upper part of the stomach is separated to form a pouch with a capacity of ≈ 30 mL. The small intestine is separated at the jejunum into a proximal biliopancreatic limb (≈40 cm, composed of the duodenum and proximal jejunum that remains in continuity with stomach) and a Roux limb (75–150 cm, beginning where jejunum is divided). The top of the Roux limb is surgically attached to the stomach pouch, and the bottom is attached to the biliopancreatic limb [7]. The illustration was obtained from [8].

RYGB alters the anatomy and physiology of the gastrointestinal tract, wherein a small gastric pouch of 15–30 mL capacity is created. The distal part of the stomach and the proximal small intestine is bypassed by attaching the distal jejunum to the small gastric pouch, creating a Roux limb (Figure 1). These surgical adaptations alter various physiologic parameters that can impact the rate and extent of drug absorption of orally administered medications in patients post-gastric bypass surgery [4]. These changes include reduced gastric volume and gastric emptying time, increased stomach pH, reduced surface area of absorption, delayed small intestinal transit time and delayed bile flow, and altered exposure to drug-metabolizing enzymes and transporters along the GI tract [5,6].

Currently, dosing decisions in patients following gastric bypass surgery generally do not consider the potential impact of surgery on systemic drug exposure of orally administered medications, which could impact safety and efficacy. This includes antimicrobial medications, which are commonly prescribed to RYGB patients who are at risk for developing a variety of infections (e.g., intra-abdominal, urinary tract, respiratory, and skin and soft tissue infections) after surgery [6]. To date, published studies investigating the impact of RYGB on oral antibiotic bioavailability are limited. A recent systematic review of the literature found only 10 published clinical studies reporting the impact of bariatric surgery on oral antibiotic absorption. The results indicated that certain classes of antibiotics have unpredictable absorption post-bariatric surgery, and this could predispose patients to antibiotic failure or toxicity [9].

Given the scarcity of data regarding potential alterations in systemic exposure post-bariatric surgery, there is a need to investigate whether altered pharmacokinetics following RYGB surgery could impact treatment outcomes in patients prescribed oral antibiotics therapy. Physiologically based pharmacokinetic (PBPK) modeling is well suited to fill the current knowledge gap.

PBPK modeling relies on an array of biologic (e.g., anatomy, physiology, genetics) and drug-specific (e.g., physicochemical, metabolic) information to simulate the disposition (ADME) characteristics of a medication after it is administered to patients and the corresponding plasma concentration–time profile. [10]. Over the past decade, PBPK modeling has become critical to drug discovery and development programs, and it is useful for assessing changes in systemic drug exposures in special patient populations, including bariatric surgery patients [10,11]. This approach, in conjunction with pharmacodynamic (PD) modeling, can be used to identify optimal dosing regimens based on target plasma concentrations and PD endpoints [12].

One of the most common infections seen in patients after bariatric surgery is community-acquired pneumonia (CAP) [13]. Azithromycin, a broad-spectrum macrolide antibiotic, is a key component of the first-line CAP treatment regimen [14]. The drug undergoes extensive distribution in the body, including white blood cells and fibroblasts. As a result, sufficiently high azithromycin concentrations are sustained at the site of infection for an extended duration. [15,16]. A total of 1500 mg of immediate-release azithromycin administered in divided doses over 3 or 5 days (e.g., 500 mg once daily for 3 days or 500 mg on day 1 followed by 250 mg on days 2–5) is the recommended regimen to treat most infections [17]. After oral administration of a suspension formulation, the absolute bioavailability is 37% [18]. Since the primary site of absorption of azithromycin is the upper small intestine [19], which is surgically bypassed in RYGB patients, there is a risk of altered drug bioavailability in patients post-bariatric surgery, depending on the type of formulation that is prescribed.

In this research, PBPK modeling was used to evaluate changes in azithromycin absorption and systemic exposure in RYGB patients following oral administration of solid and liquid formulations. Model predictions were used to assess the potential impact on azithromycin antimicrobial efficacy.

## 2. Materials and Methods

PBPK modeling was performed using the Simcyp Simulator (Version 20, Certara (Simcyp Division), Sheffield, UK).

### 2.1. Verification of RYGB Surgery Population in Simcyp

The RYGB surgery population was adopted from the literature [11,20] by modifying the morbidly obese population (BMI > 40 Kg/m^2^) available in the Simcyp library. Specific anatomic and physiologic parameters that are altered post-surgery were incorporated into the population (Table S1). These include reduced gastric capacity, altered gastric emptying, bypass of the duodenum, and changes in the intestinal expression of CYP3A4. 

The developed RYGB surgical population was verified using two probe substrates, atorvastatin and midazolam, by comparing the PBPK model predicted systemic exposure to reported clinical data. These were the same probe medications used to verify the RYGB population previously [11,20]. 

#### 2.1.1. Population Verification: Atorvastatin

Atorvastatin was selected because it was previously used to verify the RYGB surgical population [21]. A substrate profile (Table 1) of atorvastatin was created based on physiochemical properties and pharmacokinetic parameters [22,23,24,25,26,27,28,29]. Absorption was modeled using Simcyp’s ADAM module. CYP3A4 is the main metabolizing enzyme for atorvastatin, with additional metabolism mediated by CYP2C8. UGT1A1- and 1A3-mediated glucuronidation of atorvastatin acid were also considered as part of model development. Atorvastatin also undergoes non-enzymatic conversion to lactone conversion in the stomach, and the stomach degradation function in Simcyp was used to mimic acid-to-lactone conversion. Atorvastatin is a substrate for membrane transporters P-gp and OATP (1B1, 1B3, 2B).

The developed atorvastatin PBPK model was verified in healthy volunteer, pre-RYGB (morbidly obese), and post-RYGB populations. Verification of the developed atorvastatin PBPK model was performed by visual predictive check, where model simulated plasma-concentration–time profiles for each population were compared with observed data from clinical data reported in the literature [30,31,32]. The data from those publications were obtained by digitizing the plasma profiles using WebPlotDigitizer [33]. Additionally, fold-error was calculated as the ratio of model predicted and observed values for pharmacokinetic parameters, C_max_ and AUC, as a part of the model verification process. Table S2 presents the trial designs used in the simulations, which were matched to referenced clinical studies. 

#### 2.1.2. Population Verification: Midazolam

A midazolam compound file is included in the Simcyp^®^ library and was adopted for this investigation without any further modification. Midazolam was selected as a second probe to verify the RYGB surgery population by comparing PBPK model predictions against published clinical data in morbidly obese patients (BMI 51.9 ± 12.1 Kg/m^2^) pre- and post-bariatric surgery [34]. Simulations (based on the clinical study protocol) were performed on 120 subjects (10 trials of 12 subjects each), age range 37–55 years, 75% females, with sample collection for 12 h after dosing midazolam (2 mg oral solution, fasted). Model verification was assessed as described previously.

### 2.2. Azithromycin Model Development and Verification in Healthy Volunteer Population in Simcyp^®^

A compound profile (Table 2) for azithromycin was created based on physiochemical properties and pharmacokinetic parameters [35,36,37,38,39,40,41]. Oral absorption was characterized using the ADAM module, and intrinsic solubility was predicted using the Simcyp^®^ prediction toolbox. Likewise, azithromycin biliary clearance was estimated using the Simcyp^®^ retrograde model. The developed PBPK model was then verified against clinical data after an IV and oral administration (tablet and suspension) in healthy subjects.

The model was next used to simulate azithromycin plasma levels over time after both intravenous IV and oral dosing (tablet and suspension formulations) according to the trial designs in Table S3. The results were compared to published clinical data [38,41,42,43] according to the verification criteria described previously. 

### 2.3. Azithromycin PBPK Modeling in Pre- and Post-Gastric Bypass Populations

The azithromycin PBPK model was used to identify differences in systemic drug exposure between pre- and post-RYGB surgery. Simulations were first carried out for single-dose azithromycin (500 mg tablet), and the results were compared to a published clinical study evaluating azithromycin pharmacokinetics in patients before and after surgery [44]. Model predictions were also performed for multiple dose regimens for both tablet and suspension formulations to assess differences in relative bioavailability in RYGB patients. 

Simulations (based on the clinical study protocol) were performed on 140 subjects (10 trials of 14 subjects each), age range 18–60 years, 100% females, with sample collection for 24 h after dosing azithromycin. Besides single dose (500 mg), two multiple dosing regimens were simulated: 3-day (500 mg/day) and 5-day (500 mg day 1, then 250 mg/day on days 2–5). These are the recommended dosing regimens for azithromycin [17].

### 2.4. Integration of PBPK Modeling Results with Azithromycin Antimicrobial Pharmacodynamics

Azithromycin is active against *Haemophilus influenzae* and *Moraxella catarrhalis*, two common pathogens causing CAP. Azithromycin exhibits concentration-dependent with time-dependency activity against these pathogens, which can be described as the ratio of unbound 24 **h** AUC (AUC_24_) to the minimum inhibitory concentration (MIC). To simulate the PD (AUC_24_/MIC) of azithromycin, we utilized the MIC_50,_ as this was the median MIC across a large population of *H. influenzae* and *M. catarrhalis* isolates [45]. The MIC_50_ for *M. catarrhalis* was 0.06 mg/L, and it was 1 mg/L for *H. influenzae* [46,47]. We additionally tested *H. influenzae* at a MIC of 0.5 mg/L because this MIC is reported in nearly 40% of the population. The MIC_50_ was then utilized to target an AUC_24_/MIC ratio required for clinical success of >5 for *H. influenzae*, and >25 against *M. catarrhalis,* as previously described [48,49].

## 3. Results

### 3.1. Verification of RYGB Surgery Population in Simcyp

#### 3.1.1. Population Verification: Atorvastatin

Published clinical studies demonstrated altered bioavailability of atorvastatin and midazolam after bariatric (RYGB) surgery [32,34], and these medications were used to verify the RYGB surgery population used in this investigation. 

The first step of the modeling strategy was to develop a PBPK model of atorvastatin and verify it in the Simcyp^®^ Healthy Volunteer population. The model-predicted plasma concentration vs. time profiles with overlayed clinical data are presented in Figure S1. The figure demonstrates good agreement between the simulated mean profile and published clinical data. The calculated fold errors for C_max_ and AUC were between 0.80 and 1.13, indicating good agreement between PBPK-model-predicted and clinically observed data. These results demonstrate that the model successfully captured the drug behavior in healthy volunteers.

Next, the atorvastatin model was used to verify the RYGB surgery population that was adapted from Darwich et al. [11]. In a clinical study, gastric bypass surgery was found to significantly impact atorvastatin oral bioavailability, although there was large intersubject variability in the direction (↑ or ↓) and magnitude of the effect [32]. Atorvastatin is a CYP3A4/5 and P-gp substrate, and the results were attributed to intersubject differences in presystemic metabolism pre-surgery. PBPK model predictions (Table 3) were in good agreement with the results observed clinically based on fold errors of model predictions (the published study did not report plasma concentration–time profiles, so visual predictive checks could not be performed). Additionally, the simulated dose-dependent changes in bioavailability after surgery were consistent with clinical observations. Whereas AUC increased post-surgery for the lowest dose tested (20 mg), exposure was lower post-surgery for the 40 and 80 mg doses. The performance of the PBPK modeling in this study was consistent with RYGB population verification results reported previously [21].

#### 3.1.2. Population Verification: Midazolam

The RYGB surgery population was next verified with midazolam, another CYP 3A4 substrate, using the compound file available in the Simcyp^®^ Simulator. When an oral solution (2 mg) of midazolam was administered to female RYGB patients post-surgery, the rate of absorption was significantly increased with higher C_max_ and shorter T_max_ compared to pharmacokinetic testing in subjects before surgery [34]. However, RYGB did not impact the extent (AUC) of midazolam absorption. PBPK model simulations successfully captured these results, as illustrated in Figure S2. Visual inspection of the plasma concentration–time profiles showed good agreement between simulated and observed midazolam concentrations pre- and post-surgery, and the calculated fold errors of systemic exposure parameters (range 0.73 to 1.25) supported model verification pre-and post-surgery. The PBPK model successfully predicted the changes in C_max_ and T_max_ observed in the clinical study. 

### 3.2. Azithromycin Model Development and Verification in Healthy Volunteer Population in Simcyp^®^

Model simulations with the azithromycin PBPK model were initially performed for an intravenous infusion (1 or 2 g infused over two hours). The predicted systemic exposure metrics (AUC and C_max_) were in close agreement with the clinically data, with fold errors near 1.0 (Table 4). 

**Figure 2:**
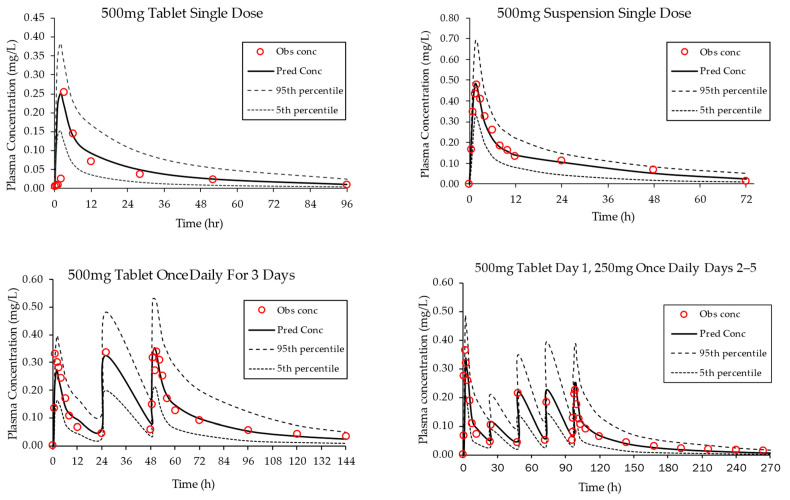
Verification of the azithromycin PBPK model in a healthy volunteer population. Plasma concentration vs. time profiles were simulated for both single and multiple oral doses and overlayed with observed clinical data [41,42,43]. Included in each plot are the model-predicted mean curve and the 5th percentile and 95th percentiles. The open circles represent clinically observed data.

Once the PBPK model was verified for intravenous dosing, the model underwent additional evaluation for oral administration, testing different single- and multiple-dose regimens for tablet formulation. As illustrated in Figure 2, simulated plasma concentration vs. time profiles aligned closely with clinical observations. Likewise, the PBPK model predicted that C_max_ and AUC values were in close agreement with reported values for all regimens tested (fold error range 0.86–1.01, Table 4). The model also predicted the pharmacokinetics of a suspension formulation of azithromycin (500 mg, single dose).

Thus, the oral pharmacokinetics of azithromycin for different regimens of tablet and suspension formulations were successfully predicted using the developed PBPK model (Figure 2), supporting its potential application to assess the pharmacokinetics of azithromycin in an RYGB surgery population. 

### 3.3. PBPK Modeling of Azithromycin in Pre- (Morbidly Obese) and Post-Gastric Bypass Populations

**Figure 3:**
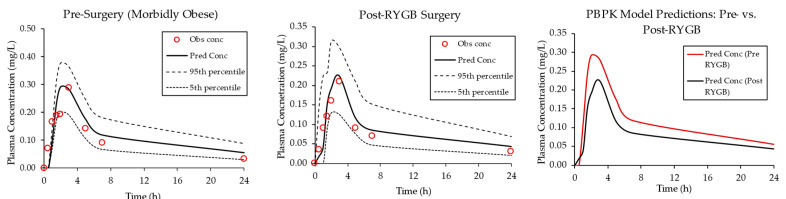
PBPK model-simulated concentration–time profiles for azithromycin in RYGB patients before (left panel) and after surgery (middle panel). Included in each plot are the model-predicted mean curve and the 5th percentile and 95th percentiles. The open circles represent clinically observed data [44]. The right panel compares the simulated mean profile pre- and post-surgery.

The azithromycin PBPK model was then used to simulate plasma concentration–time profile pre- (morbidly obese) and post-RYGB surgery after oral administration. A published clinical study reported reduced azithromycin C_max_ and AUC after bariatric surgery compared to baseline (pre-surgery) following the administration of a single 500 mg dose [44]. PBPK modeling was able to capture these observations, as demonstrated in Figure 3 and Table 5. 

The results show that the PBPK model successfully captured the pharmacokinetic profile of azithromycin in pre- and post-RYGB populations. Fold errors for AUC and C_max_ were close to 1.0 (range 0.88 to 1.22), which indicates that the model successfully predicted the pharmacokinetic parameters compared to the observed data. A significant decrease in the extent of absorption was observed for a single dose of azithromycin post-surgery. The predicted AUC for the post-surgical group was 32% lower (1.7 ± 0.51 mg-h/L vs. 2.5 ± 0.63 mg-h/L) than the pre-surgical (morbidly obese) group, and this was in close agreement with the published clinical study that also reported 32% lower AUC in RYGB subjects after surgery. Likewise, azithromycin peak plasma concentrations were reduced after RYGB surgery (Table 6). 

The PBPK model was then used to predict the steady-state plasma concentration profile of azithromycin (tablet formulation) for the two clinically indicated dosing regimens (500 mg once daily for 3 days; 500 mg on day 1, 250 mg on days 2–5) in pre- and post-surgery populations. The results are provided in Figure S3 and point to altered bioavailability in RYGB patients. The predicted azithromycin steady-state AUC after surgery was 30% lower (22.5 ± 4.20 mg·h/L vs 15.7 ± 5.36 mg·h/L) compared to simulations in a morbidly obese population (pre-surgery) group for the three-day regimen. Similar results were seen for the five-day regimen, where the model predicted AUC for post-surgery was 28% lower (19.98 ± 3.79 mg·h/L vs. 14.47 ± 3.42 mg·h/L lower than the pre-surgical group. A statistically significant (*p* < 0.05) decrease in PBPK model-predicted overall exposure was observed at a steady state of azithromycin post-surgery. 

Lastly, the PBPK model simulations in the post-RYGB surgery population were repeated for an oral suspension formulation for single and multiple dosing. Table 6 compares systemic exposure predictions relative to the tablet formulation. The predicted AUC following single-dose administration of the suspension was three times higher than the tablet. The magnitude of the difference was reduced (1.4–1.5-fold higher AUC) for multiple-dose regimens. The lower relative bioavailability of the tablet formulation in this population may impact clinical efficacy.

### 3.4. Integration of PBPK Modeling Results with Azithromycin Antimicrobial Pharmacodynamics

To evaluate the potential impact of altered systemic exposure on azithromycin antimicrobial efficacy post-bariatric surgery, steady-state AUC_24_/MIC ratios were calculated for relevant pathogens of respiratory infections, *H. influenzae* and *M. catarrhalis,* in pre- (morbidly obese) and post-surgical populations for three-day and five-day oral regimens (Table 7). The data suggest that, for the tablet formulation, azithromycin systemic exposure was subinhibitory (below the threshold ratio of 5) for *H. influenzae* with a MIC of 0.5 and 1 mg/L after RYGB surgery. The tablet formulation also failed to meet the threshold in the pre-surgery population for the *H. influenzae* with the higher MIC of 1 mg/L. Conversely, the suspension formulation, AUC_24_/MIC results indicated that the target threshold was reached for *H. influenzae* MIC of 0.5 mg/L for both three-day and five-day dosing regimens in gastric bypass patients. However, for *H. influenzae* MIC of 1 mg/L, the threshold ratio was not attained. For *M. catarrhalis*, pharmacodynamic endpoints were above the threshold for the tablet and suspension formulation, although the AUC_24_/MIC was twofold lower post-bariatric surgery for the tablet formulation.

## 4. Discussion

RYGB surgery limits an individual’s intake and absorption capacity by combining a small gastric reservoir and bypass of the upper small intestine, thereby promoting weight loss. However, anatomical alterations caused by this procedure may interfere with oral drug absorption, thereby altering pharmacokinetics in patients after RYGB. Specific changes that may influence drug dissolution and absorption post-surgery include increased gastric pH, reduced gastric emptying and intestinal transit time, dissociation of bile salt delivery, and reduced intestinal metabolism and efflux [50]. 

This investigation applied an in silico approach to evaluate changes in systemic exposure of azithromycin after oral administration post-RYGB using PBPK modeling and simulation with the Simcyp Simulator^®^. An RYGB surgery population, first reported by Darwich et al. [11], was adapted and used to simulate the plasma concentration vs. time profile for azithromycin and to compare the results to the Simcyp morbidly obese population that represented patients pre-surgery. The RYGB surgery population was first verified against published clinical data on atorvastatin and midazolam, medications that were previously adopted for this purpose [19,20]. The verification process produced results that were reflective of a well-characterized PBPK model for studying alterations in drug disposition after bypass surgery by accounting for alterations in various intrinsic parameters. For atorvastatin, the pre- to post-surgery trends in oral exposure were highly variable, consistent with clinical data, and attributed to differences in CYP and P-gp activity that were influenced by intersubject variability combined with the surgical alterations in these processes. PBPK modeling predicted a considerable increase in the rate of midazolam absorption (↓ T_max_), but that had no impact of overall exposure (AUC) after RYGB surgery. These findings matched published clinical findings.

The azithromycin PBPK models were able to capture the plasma concentration–time profiles following oral dosing to healthy volunteers as well as morbidly obese (pre-surgery) and RYGB subjects. The PBPK model developed in this study was first extensively verified using the healthy volunteer population in Simcyp^®^ for intravenous and oral administration. The model predicted the systemic profile of azithromycin for both single- and multiple-dosing regimens for a tablet formulation. 

Model predictions pointed to reduced bioavailability of azithromycin (tablet formulation, single dose) after RYGB surgery, with a mean AUC ratio (post-/pre-surgery) of 0.67. This difference, which was consistent published clinical findings, was attributed to a reduced fraction of azithromycin dose absorbed by the intestine (f_a_, 0.71 ± 0.03 to 0.56 ± 0.04), leading to increased oral clearance (Cl/F, 174.4 ± 63.7 L/h to 262.6 ± 99.5 L/h) after surgery. Similar findings were obtained for multiple dose simulations (tablet formulation) with an AUC ratio (post/pre-surgery) of ≈0.7. 

Azithromycin is classified a BCS class 2 (high permeability/low solubility) medication. The rate-limiting step to oral absorption is dissolution in the GI fluids, although absorption of azithromycin post-surgery may be compromised by the impact of bypassing the upper small intestine, which is the primary site of absorption for azithromycin in the GI tract. An immediate-release tablet formulation requires disintegration and subsequent dissolution as prerequisites for absorption. RYBG surgery causes significant reduction in gastric retention time, and a tablet dosage form of azithromycin would likely travel at a similar or slightly slower speed than the GI fluids transiting the stomach pouch to the distal jejunum (due to bypassing upper small intestine post-surgery), where water is rapidly absorbed. This would likely have a negative effect on disintegration of the tablet and subsequent drug dissolution. Consequently, the shortened length of the intestine and reduced gastric retention likely results in altered dissolution and lower oral bioavailability when administered as a tablet formulation to patients after bariatric surgery [51,52,53].

A suspension formulation, by contrast, does not have the same limitations as a solid dosage form, and the impact of RYGB surgery would be expected to be minimized with this type of formulation.

This was demonstrated by PBPK modeling where, compared to a suspension formulation, the bioavailability of the tablet formulation was reduced in RYGB patients. Additionally, plasma exposure (AUC) of the suspension formulation post-surgery was comparable to levels observed in patients treated with a tablet formulation before bariatric surgery. It has been proposed that healthcare providers treating patients who have undergone bariatric surgery prescribe liquid formulations, if available [54]. The results observed with azithromycin in the present study support this general recommendation of switching from solid to liquid formulations in RYGB patients after surgery.

Ultimately, an important question to answer is whether altered pharmacokinetics after RYGB surgery potentially impact the safety and efficacy of medications, including antibiotics. For azithromycin, the relevant PK/PD marker is the AUC_24_/MIC, and PBPK model simulation results were used to evaluate whether threshold values of this endpoint were achieved for the commonly prescribed three-day and five-day dosing regimens. Despite reduced systemic exposure post-surgery, target threshold values against *M. catarhallis* (AUC_24_/MIC > 25) were achieved for both tablet and suspension formulations. Conversely, the tablet formulation failed to reach the PD endpoint (AUC_24_/MIC > 5) for *H. influenzae* post-surgery. However, the suspension formulation was able to reach the AUC_24_/MIC target against *H. influenzae* with a MIC of 0.5 mg/L, but still not for isolates with a MIC of 1 mg/L. Interestingly, the tablet formulation also failed to reach the threshold in the pre-surgery (morbidly obese) population against *H. influenzae* with a MIC of 1 mg/L and may reflect differences in pharmacokinetics from healthy weight subjects. These findings suggest that reduced bioavailability increases the risk of therapeutic failure for azithromycin against infections caused by *H. influenzae* and support the general recommendation that liquid formulations such as suspensions may be a better alternative for treating respiratory infections in the gastric bypass population.

## 5. Conclusions

PBPK modeling and simulation provide a framework for theoretical exploration of the impact of altered anatomic and physiologic mechanisms on systemic drug exposure of orally administered medications following gastric bypass surgery. Platforms such as the Simcyp^®^ Simulator provide a framework for studying mechanisms involved in altering oral drug exposure after RYGB surgery, such as the interplay among drug dissolution, absorption, and presystemic metabolism along with physicochemical and formulation properties, as well as the subsequent impact on the plasma profile of medications in RYGB patients. PBPK modeling can be used to predict the effect of food- and acid-reducing agents on systemic exposure in RYGB patients, as well as evaluate other potential drug–drug interactions. This, in turn, provides a framework for pharmacotherapeutic drug optimization by identifying safe and effective dosing regimens in the special patient population.

## Figures and Tables

**Table 1:** Input parameters to create atorvastatin compound profile in Simcyp^®^.

Parameter	Value	Reference
**Physicochemical Properties**		
Molecular weight	546 g/mol	[22]
Log P	4.2	[22]
pKa	4.46	[22]
Blood/plasma ratio	0.61	[25]
Fraction unbound in plasma	0.027	[25]
**Absorption**		
Absorption model	ADAM	[24]
Caco-2 (pH 6.4:7.5) (10^−6^ cm/s)	4.9	
**Dissolution:**		
Aqueous solubility @ pH 6.0 (mg/mL)	1.23	[23]
**Distribution**		
Distribution model	Full PBPK model	
Prediction method	Method 2 (Rodgers and Rowland)	
Kp scalar	2 (optimized)	
**Metabolism/Elimination**		
**Clearance Type**	Enzyme kinetics	[26,27,28]
CYP3A4: para-OH		
ClINT (μL min^−1^ mg^−1^ protein)	35.5 ± 48.1	
CYP3A4: ortho-OH		
ClINT (μL min^−1^ mg^−1^ protein)	45.8 ± 59.1	
CYP2C8		
ClINT (μL min^−1^ mg^−1^ protein)	10.5	
Additional HLM		
ClINT (μL min^−1^ mg^−1^ protein)	65	
UGT1A1		
ClINT (μL min^−1^ mg^−1^ protein)	5.23	
UGT1A3		
ClINT (μL min^−1^ mg^−1^ protein)	6.2	
**Transport**		
P-gp (apical efflux, small intestine)		[24]
Jmax (pmol/min)	141 ± 11	
Km (μM)	115 ± 19	
OATP1B1 (sinusoidal uptake, liver)		[29]
Jmax (pmol/min/10^6^ hepatocytes)	25	
Km (μM)	0.77	
OATP1B3 (sinusoidal uptake, liver)		
Jmax (pmol/min/10^6^ hepatocytes)	25	
Km (μM)	0.73	
OATP2B1 (sinusoidal uptake, liver)		
Jmax (pmol/min/106 hepatocytes)	24.27	
Km (μM)	2.84	

**Table 2:** Input parameters to create azithromycin compound profile in Simcyp^®^.

Parameter	Value	Reference
**Physicochemical Properties**		
Molecular weight	749.12 g/mol	[35]
Log P	4.02	[35]
pKa	8.5	[35]
Blood/plasma ratio	1	Assumed
Fraction unbound in plasma	0.69 (bound to Alpha-1 acid glycoprotein)	[35]
**Absorption**		
Absorption model	ADAM	
PeffCaco-2 (pH 6.4:7.5) (10^−6^ cm/s)	3.59	[36]
**Dissolution**		
Intrinsic solubility ((mg/mL)	0.029	Predicted
Melting point (°C)	114.8	[37]
**Distribution**		
Distribution model	Full PBPK model	
Prediction method	Method 2 (Rodgers and Rowland)	
**Metabolism/Elimination**		
Clearance type	Enzyme kinetics	
Biliary intrinsic clearance (µL/min/10^6^)	9.25 (Calculated using retrograde model)	
Cl_IV_ (L/h)	46.5	[38]
Cl_R_ (L/h)	8.67	[39]
**Transport**		
P-gp (apical efflux, small intestine)		
Jmax (nmol/min)	90.7	[40]
Km (µM)	11.3	

**Table 3:** Verification of atorvastatin compound file in pre- (morbidly obese) and post-RYGB surgery populations in the Simcyp^®^ Simulator.

Dose (mg)	Atorvastatin AUC (ng·h/mL)					
	Pre-Surgery (Morbidly Obese)			Post-Surgery (RYGB Surgery Population)		
	Obs ^a^	Pred ^b^	FE ^c^	Obs ^a^	Pred ^b^	FE ^c^
20	18.5	20.7	1.12	28	25.3	0.9
40	100.5	98.3	0.98	64	53.1	0.83
80	175.0	171.96	0.98	84	83.6	0.99
**Dose** **(mg)**	**Atorvastatin C_max_ (ng /mL)**					
	**Pre-Surgery**			**Post-Surgery**		
	Obs ^a^	Pred ^b^	FE ^c^	Obs ^b^	Pred ^b^	FE ^c^
20	5.0	3.4	0.67	5.0	4.2	0.84
40	22.0	16.6	0.75	17.5	8.3	0.47
80	70.0	26.9	0.38	23.0	13.2	0.58
**Dose** **(mg)**	**Atorvastatin T_max_ (h)**					
	**Pre-Surgery**			**Post-Surgery**		
	Obs ^a^	Pred ^b^	FE ^c^	Obs ^b^	Pred ^b^	FE ^c^
20	0.9	1.5	1.56	1.1	1.4	1.27
40	1.1	2.0	1.81	2.3	1.4	0.61
80	3.8	2.0	0.53	2.4	1.4	0.58

^a^ Observed parameter estimate reported in the literature [32]. ^b^ Predicted parameter estimate predicted by PBPK modeling using Simcyp^®^. ^c^ Fold error- ratio of [predicted]/[observed] values.

**Table 4:** Verification of azithromycin compound file in healthy volunteer populations in the Simcyp^®^ Simulator.

Dosing Regimen	Parameter	Observed	Predicted	Fold Error	Reference
IV infusion (1 g infused over 2 h)	C_max_ (mg/L)	3.1 ± 0.4	3.2 ± 0.4	1.03	[38]
	AUC (mg·h/L)	23.0 ± 4	23.3 ± 4.9	1.01	
IV infusion (2 g infused over 2 h)	C_max_ (mg/L)	6.9 ± 2.0	6.4 ± 0.8	0.94	
	AUC (mg·h/L)	46.0 ± 9.0	46.5 ± 9.8	1.01	
Single oral dose (500 mg tablet)	C_max_ (mg/L)	0.30 ± 0.34	0.26 ± 0.07	0.86	[41]
	AUC (mg·h/L)	4.4 ± 1.9	4.4 ± 1.8	1.01	
500 mg (tablet) Once/day for 3 days	AUC (mg·h/L)	19.4 ± 7.9	17.2 ± 3.5	0.89	[42]
500 mg (tablet) day 1, then 250 mg days 2–5	AUC (mg·h/L)	15.9 ± 4.8	14.5 ± 3.1	0.91	[42]
Single oral dose (500 mg suspension)	C_max_ (mg/L)	0.49 ± 0.13	0.49 ± 0.11	1.01	[43]
	AUC (mg·h/L)	7.57 ± 2.63	6.45 ± 2.11	0.85	

Observed parameter estimate reported in the literature. References are listed in the Table. Predicted parameter estimate predicted by PBPK model using Simcyp^®^ Simulator. Fold-error = [predicted]/[observed] values.

**Table 5:** Verification of azithromycin compound file in pre- (morbidly obese) and post-RYGB surgery populations in the Simcyp^®^ Simulator.

Parameter	Pre-Surgery			Post-Surgery		
	Observed	Predicted	Fold Error	Observed	Predicted	Fold Error
C_max_ (mg/L)	0.36 ± 0.2	0.33 ± 0.06	0.91	0.26 ± 0.1	0.23 ± 0.05	0.88
AUC (mg·h/L)	2.1 ± 0.8	2.5 ± 0.63	1.22	1.4 ± 0.5	1.7 ± 0.51	1.20
T_max_ (h)	2.4 ± 1.2	2.3 ± 0.4	0.96	2.1 ± 0.1	2.2 ± 0.5	1.01

Observed parameter estimate reported in the literature [39]. Predicted parameter estimate predicted by PBPK model using Simcyp^®^ Simulator. Fold error = [predicted]/[observed] values.

**Table 6:** PBPK model assessment of relative bioavailability of azithromycin tablet and suspension formulations after RYGB surgery using the Simcyp^®^ Simulator.

Dosing Regimen	C_max_ (mg/L)		AUC_0-t_ (mg·h/L)		Relative Bioavailability (Tablet: Suspension)
	Tablet	Suspension	Tablet	Suspension	
500 mg single dose	0.23 ± 0.05	0.41 ± 0.09	1.69 ± 0.51	5.16 ± 1.77	0.33
500 mg QD for 3 days	0.28 ± 0.07	0.47 ± 0.13	15.7 ± 5.36	22.0 ± 5.16	0.71
500 mg day 1250 mg day 2–5	0.28 ± 0.06	0.49 ± 0.10	14.5 ± 3.42	22.2 ± 4.47	0.65

**Table 7:** Assessment of antimicrobial PK/PD target attainment pre- and post-RYGB surgery: comparison of azithromycin tablet and suspension formulations.

500 mg Once Daily For 3 Days									
	MIC (mg/L)	AUC_24_ (mg·h/L) Day 3				AUC_24_/MIC			
		Pre-Surgery		Post-Surgery		Pre-Surgery		Post-Surgery	
		Tablet	Susp	Tablet	Susp	Tablet	Susp	Tablet	Susp
*H. influenzae*	1	4.3	5.2	2.4	4.1	4.3	5.2	2.4	4.1
*H. influenzae*	0.5	4.3	5.2	2.4	4.1	8.6	10	4.8	8.2
*M. catarrhalis*	0.06	4.3	5.2	2.4	4.1	72	87	40	87
500 mg day 1; 250 mg Days 2 To 5									
	**MIC** **(mg/L)**	**AUC24 (mg·h/L) Day 5**				**AUC24/MIC**			
		**Pre-Surgery**		**Post-Surgery**		**Pre-Surgery**		**Post-Surgery**	
		**Tablet**	**Susp**	**Tablet**	**Susp**	**Tablet**	**Susp**	**Tablet**	**Susp**
*H. influenzae*	1	3.2	3.3	2.2	3.1	3.2	3.3	2.2	3.1
*H. influenzae*	0.5	3.2	3.3	2.2	3.1	6.4	6.6	4.4	6.2
*M. catarrhalis*	0.06	3.2	3.3	2.2	3.1	53	55	37	52

Target AUC_24_/MIC_90_ ratios were >5 against *H. influenzae*, and >25 against *M. catarrhalis*. Dosing regimens that did not reach the PD target are shaded in red.

## Data Availability

The data presented in this study are available on request from the corresponding author.

## Supplementary Materials

The following supporting information can be downloaded at https://www.mdpi.com/article/10.3390/pharmaceutics15112520/s1. Table S1: Parameter modifications to a Simcyp morbidly obese population in Simcyp to create an RYGB surgery population [11]. Table S2: Study design for Simcyp^®^ simulations of atorvastatin in pre- and post-surgical populations. Table S3: Study design for Simcyp^®^ simulations of azithromycin in healthy volunteers. Figure S1. Verification of atorvastatin compound file in a healthy volunteer population in the Simcyp^®^ Simulator. Figure S2. Verification of an RYGB surgical population using midazolam in the Simcyp^®^ Simulator. Figure S3. PBPK model simulations of azithromycin multiple dosing regimens (tablet formulation) in pre- and post-RYGB surgery populations using the Simcyp^®^ Simulator.

## References

[1] Ogden C.L., Fryar C.D., Martin C.B., Freedman D.S., Carroll M.D., Gu Q., Hales C.M. (2020). Trends in obesity prevalence by race and hispanic origin—1999–2000 to 2017–2018. JAMA.

[2] Magouliotis D.E., Tasiopoulou V.S., Sioka E., Chatedaki C., Zacharoulis D. (2017). Impact of bariatric surgery on metabolic and gut microbiota profile: A systematic review and meta-analysis. Obes. Surg..

[3] Mason E.E., Ito C. (1967). Gastric bypass in obesity. Surg. Clin. N. Am..

[4] Maclellan W.C., Johnson J.M. (2021). Laparoscopic gastric bypass: Still the gold standard?. Surg. Clin. N. Am..

[5] Darwich A.S., Henderson K., Burgin A., Ward N., Whittam J., Ammori B.J., Ashcroft D.M., Rostami-Hodjegan A. (2012). Trends in oral drug bioavailability following bariatric surgery: Examining the variable extent of impact on exposure of different drug classes. Br. J. Clin. Pharmacol..

[6] Ghobadi C., Johnson T.N., Aarabi M., Almond L.M., Allabi A.C., Rowland-Yeo K., Jamei M., Rostami-Hodjegan A. (2011). Application of a systems approach to the bottom-up assessment of pharmacokinetics in obese patients expected variations in clearance. Clin. Pharmacokinet..

[7] Mitchell B.G., Gupta N. (2023). Roux-en-Y Gastric Bypass. StatPearls.

[8] Levine J.W., Feng J., Feng D.P., Melvin W.V. (2017). Perioperative patient care involved with robotic-assisted bariatric surgery. Ann. Laparosc. Endosc. Surg..

[9] Anvari S., Lee Y., Lam M., Doumouras A.G., Hong D. (2020). The effect of bariatric surgery on oral antibiotic absorption: A systematic review. Obes. Surg..

[10] Shardlow C.E., Generaux G.T., Patel A.H., Tai G., Tran T., Bloomer J.C. (2013). Impact of physiologically based pharmacokinetic modeling and simulation in drug development. Drug Metab. Dispos..

[11] Darwich A.S., Pade D., Ammori B.J., Jamei M., Ashcroft D.D., Rostami-Hodjegan A. (2012). A mechanistic pharmacokinetic model to assess modified oral drug bioavailability post bariatric surgery in morbidly obese patients: Interplay between CYP3A gut wall metabolism, permeability and dissolution. J. Pharm. Pharmacol..

[12] Zhuang X., Lu C. (2016). PBPK modeling and simulation in drug research and development. Acta Pharm. Sin. B.

[13] Goto T., Hirayama A., Faridi M.K., Camargo C.A., Hasegawa K. (2017). Association of bariatric surgery with risk of infectious diseases: A self-controlled case series analysis. Clin. Infect. Dis..

[14] Metlay J.P., Waterer G.W., Long A.C., Anzueto A., Brozek J., Crothers K., Cooley L.A., Dean N.C., Fine M.J., Flanders S.A. (2019). Diagnosis and treatment of adults with community-acquired pneumonia. an official clinical practice guideline of the American Thoracic Society and Infectious Diseases Society of America. Am. J. Respir. Crit. Care Med..

[15] Blumer J.L. (2005). Evolution of a new drug formulation: The rationale for high-dose, short-course therapy with azithromycin. Int. J. Antimicrob. Agents.

[16] Liu P., Allaudeen H., Chandra R., Phillips K., Jungnik A., Breen J.D., Sharma A. (2007). Comparative pharmacokinetics of azithromycin in serum and white blood cells of healthy subjects receiving a single-dose extended-release regimen versus a 3-day immediate-release regimen. Antimicrob. Agents Chemother..

[17] Pfizer Labs (1991). Zithromax [Package Insert].

[18] Foulds G., Shepard R.M., Johnson R.B. (1990). The pharmacokinetics of azithromycin in human serum and tissues. J. Antimicrob. Chemother..

[19] Chandra R., Liu P., Breen J.D., Fisher J., Xie C., LaBadie R., Benner R.J., Benincosa L.J., Sharma A. (2007). Clinical pharmacokinetics and gastrointestinal tolerability of a novel extended-release microsphere formulation of azithromycin. Clin. Pharmacokinet..

[20] Chen K.F., Chan L.N., Lin Y.S. (2020). PBPK modeling of CYP3A and P-gp substrates to predict drug-drug interactions in patients undergoing Roux-en-Y gastric bypass surgery. J. Pharmacokinet. Pharmacodyn..

[21] Darwich A.S., Pade D., Rowland-Yeo K., Jamei M., Asberg A., Christensen H., Ashcroft D.M., Rostami-Hodjegan A. (2013). Evaluation of an in silico PBPK post-bariatric surgery model through simulating oral drug bioavailability of atorvastatin and cyclosporine. CPT Pharmacomet. Syst. Pharmacol..

[22] Lennernas H. (2003). Clinical pharmacokinetics of atorvastatin. Clin. Pharmacokinet..

[23] Morse B.L., Alberts J.J., Posada M.M., Rehmel J., Kolur A., Tham L.S., Loghin C., Hillgren K.M., Hall S.D., Dickinson G.L. (2019). Physiologically-based pharmacokinetic modeling of atorvastatin incorporating delayed gastric emptying and acid-to-lactone conversion. CPT Pharmacomet. Syst. Pharmacol..

[24] Wu X., Whitfield L.R., Stewart B.H. (2000). Atorvastatin transport in the Caco-2 cell model: Contributions of P-glycoprotein and the proton-monocarboxylic acid co-transporter. Pharm. Res..

[25] Watanabe T., Kusuhara H., Maeda K., Kanamaru H., Saito Y., Hu Z., Sugiyama Y. (2010). Investigation of the rate-determining process in the hepatic elimination of HMG- coa reductase inhibitors in rats and humans. Drug Metab. Dispos..

[26] Jacobsen B.K., Sodner A., Kirchner G., Sewing K.F., Kollman P.A., Benet L.Z., Christians U. (2000). Lactonization is the critical first step in the disposition of 3-hydroxy3- methylgultaryl-co A reductase inhibitor atorvastatin. Drug Metab. Dispos..

[27] Prueksaritanont T., Subramanian R., Fang X., Bennett M.A., Qiu Y., Lin J.H., Pearson P.G., Baillie T.A. (2002). Glucuronidation of statins in animals and humans: A novel mechanism of statin lactonization. Drug Metab. Dispos..

[28] Schirris T.J.J., Ritschel T., Bilos A., Smeitink J.A.M., Russel F.G.M. (2015). Statin lactonization by uridine 5′-diphosphoglucuronosyltransferases (UGTs). Mol. Pharm..

[29] Karlgren M., Vildhede A., Norinder U., Wisniewski J., Kimoto E., Lai Y., Haglund U., Artursson P. (2012). classification of inhibitors of hepatic organic anion transporting polypeptides (OATPs): Influence of protein expression on drug drug interaction. J. Med. Chem..

[30] Lau Y.Y., Huang Y., Frassetto L., Benet L.Z. (2006). Effect of OATP1B transporter inhibition on the pharmacokinetics of atorvastatin in healthy volunteers. Clin. Pharmacol. Ther..

[31] Bullman J., Nicholls A.N., Van Landingham K., Fleck R., Vuong A., Miller J., Alexander S., Messenheimer J. (2011). Effects of lamotrigine and phenytoin on the pharmacokinetics of atorvastatin in healthy volunteers. Epilepsia.

[32] Skottheim I.B., Stormark K., Christensen H., Jakobsen G.S., Hjelmesæth J., Jenssen T., Reubsaet J.L.E., Sandbu R., Asberg A. (2009). Significantly altered systemic exposure to atorvastatin acid following gastric bypass surgery in morbidly obese patients. Clin. Pharmacol. Ther..

[33] Rohatgi A. (2022). WebPlotDigitizer (Version 4.6) [Computer Software]. https://automeris.io/WebPlotDigitizer.

[34] Chan L.N., Lin Y.S., Tay-Sontheimer J.C., Trawick D., Oelschlager B.K., Flum D.R., Patton K.K., Shen D.D., Horn J.R. (2015). Proximal Roux-en-Y gastric bypass alters drug absorption pattern but not systemic exposure of CYP3A4 and P-glycoprotein substrates. Pharmacotherapy.

[35] McFarland J.W., Berger C.M., Froshauer S.A., Hayashi S.F., Hecker S.J., Jaynes B.H., Jefson M.R., Kamicker B.J., Lipinski C.A., Lundy K.M. (1997). Quantitative structure-activity relationships among macrolide antibacterial agents: In vitro and in vivo potency against Pasteurella multocida. J. Med. Chem..

[36] Milić A., Mihaljević V.B., Ralić J., Bokulić A., Nožinić D., Tavčar B., Mildner B., Munić V., Malnar I., Padovan J. (2014). A comparison of in vitro ADME properties and pharmacokinetics of azithromycin and selected 15-membered ring macrolides in rodents. Eur. J. Drug Metab. Pharmacokin..

[37] Pouretedal H.R. (2014). Preparation and characterization of azithromycin nanodrug using solvent/antisolvent method. Int. Nano Lett..

[38] Luke D.R., Foulds G., Cohen S.F., Levy B. (1996). Safety, toleration, and pharmacokinetics of intravenous azithromycin. Antimicrob. Agents Chemother..

[39] Lalak N.J., Morris D.L. (1993). Azithromycin clinical pharmacokinetics. Clin. Pharmacokinet..

[40] Horita Y., Doi N. (2014). Comparative study of the effects of antituberculosis drugs and antiretroviral drugs on cytochrome P450 3A4 and P-glycoprotein. Antimicrob. Agents Chemother..

[41] Beringer P., Huynh K.M.T., Kriengkauykiat J., Bi L., Hoem N., Louie S., Han E., Nguyen T., Hsu D., Rao P.A. (2005). Absolute bioavailability and intracellular pharmacokinetics of azithromycin in patients with cystic fibrosis. Antimicrob. Agents Chemother..

[42] Amsden G.W., Nafziger A.N., Foulds G. (1999). Pharmacokinetics in serum and leukocyte exposures of oral azithromycin, 1500 milligrams, given over a 3- or 5-day period in healthy subjects. Antimicrob. Agents Chemother..

[43] Zakeri-Milani P. (2010). Pharmacokinetic study of two macrolide antibiotic oral suspensions using an optimized bioassay procedure. J. Bioequiv Availab..

[44] Padwal R.S., Ben-Eltriki M., Wang X., Langkaas L.A., Sharma A.M., Birch D.W., Karmali S., Brocks D.R. (2012). Effect of gastric bypass surgery on azithromycin oral bioavailability. J. Antimicrob. Chemother..

[45] Muto C., Liu P., Chiba K., Suwa T. (2011). Pharmacokinetic –pharmacodynamic analysis of azithromycin extended release in Japanese patients with common respiratory tract infectious disease. J. Antimicrob. Chemother..

[46] Biedenbach D.J., Jones R.N., Lewis M.T., Croco M.A.T., Barrett M.S. (1999). Comparative in vitro evaluation of dirithromycin tested against recent clinical isolates of *Haemophilus influenzae*, *Moraxella catarrhalis*, and *Streptococcus pneumoniae*, including effects of medium supplements and test conditions on MIC results. Diagn. Microbiol. Infect. Dis..

[47] SENTRY Antimicrobial Surveillance, SENTRY Public. https://sentry-mvp.jmilabs.com/app/sentry-public.

[48] Alou L., Aguilar L., Sevillano D., Gimenez M.J., Gonzalez N., Echeverria O., Torrico M., Martin J.E., Valdes L., Prieto J. (2007). Levofloxacin vs. azithromycin pharmacodynamic activity against *S. pneumoniae* and *H. influenzae* with decreased susceptibility to amoxicillin/clavulanic acid. J. Chemother..

[49] Craig W.A. (2001). Does the dose matter?. Clin. Infect. Dis..

[50] Hachon L., Decleves X., Faucher P., Carette C., Lloret-Linares C. (2017). RYGB and drug disposition: How to do better? Analysis of pharmacokinetic studies and recommendations for clinical practice. Obes. Surg..

[51] Wölnerhanssen B.K., Meyer-Gerspach A.C., Peters T., Beglinger C., Peterli R. (2016). Incretin effects, gastric emptying, and insulin responses to low oral glucose loads in patients after gastric bypass and lean and obese controls. Surg. Obes. Relat. Dis..

[52] Nguyen N.Q., Debreceni T.L., Burgstad C.M., Wishart J.M., Bellon M., Rayner C.K., Wittert G.A., Horowitz M. (2015). Effects of posture and meal volume on gastric emptying, intestinal transit, oral glucose tolerance, blood pressure, and gastrointestinal symptoms after Roux-en-Y Gastric Bypass. Obes. Surg..

[53] Brocks D.R., Ben-Eltriki M., Gabr R.Q., Padwal R.S. (2012). The effects of gastric bypass surgery on drug absorption and pharmacokinetics. Exp. Opin. Drug Metab. Toxicol..

[54] Stano S., Alam F., Wu L., Dutia R., Ng S.N., Sala M., McGinty J., Laferrère B. (2017). Effect of meal size and texture on gastric pouch emptying and glucagon-like peptide 1 after gastric bypass surgery. Surg. Obes. Relat. Dis..

