# Supplementary material for: PBPK Modeling of Azithromycin Systemic Exposure in a Roux-en-Y Gastric Bypass Surgery Patient Population
Source: Pharmaceutics. 2023 Oct 24;15(11):2520. doi: 10.3390/pharmaceutics15112520 (PMC10674169; doi:10.3390/pharmaceutics15112520)
Supplement: Supplementary file 1 [file pharmaceutics-15-02520-s001.zip › pharmaceutics-2653301-supplementary.pdf]

Table S1. Parameter modifications to Simcyp Morbidly Obese population in Simcyp to create an RYGB Surgery Population [11].

Parameter	RYGB Modification
Gastric emptying: liquids (minutes)	7; CV=45%
Gastric capacity (ml)	30
Qsec stomach (l/h)	0.059
Initial volume of stomach acid (ml)	9.9
Gastric pH	6.5
Small intestinal bypass (centimeters and/or segments)[a]	100cm (duodenum and jejunum I)
Bile exclusion (centimeters and segments)	110cm (Stomach and jejunum I)
CYP3A4 abundance (nmol/total gut)	48.3 CV:60%
CYP3A5 abundance (nmol/total gut)	18.0 CV:60%
Mean small intestinal transit time (hours)[b]	3.0; α=2.6, β=3.7
GFR estimation	MDRD

CV, coefficient of variation; MDRD, modification of diet renal disease equation; Qsec, secretion flow; RYGB, Roux-en-Y gastric bypass;

[a]Human effective permeability (*P*eff) in bypassed segments was set to zero in the Simcyp® compound file.

[b]Modeled as a Weibull function based on parameters α and β. The equation is reported in reference 11.

Table S2. Study design for Simcyp® simulations of atorvastatin in pre- and post-surgical population.

Trial Simulation Design Variable	Population				
	HV #1	HV#2	Pre- and Post-RYGB Surgery		
Number of trials	10	10	10	10	10
Number of subjects/trial	12	63	6	4	2
Age of Subjects (years)	24-48	18-55	29-63	29-63	29-63
% of females	50	20	33	100	0
Duration of sample collection (h)	24	24	12	12	12
Dosing regimen	40mg single dose	40mg QD for 7days	20mg single dose	40mg single dose	80mg single dose
Reference	27	28	29		

Table S3. Study design for Simcyp® simulations of azithromycin in healthy volunteers.

Trial Simulation Design Variable	Intravenous Infusion	Oral (Tablet Formulation)		Oral (Suspension Formulation)
		Single Dose	Multiple Dose	
Number of trials	10	10	10	10
Number of subjects/trial	8	12	12	24
Age of Subjects (years)	18-43	22-39	30-45	21-31
% of females	0	33	50	0
Duration of sample collection (h)	240	96	288	72
Dosing regimen	1mg and 2 mg infused over 2 hours	500mg single dose	500mg once daily for 3 days; 500mg day 1, 250mg days 2-5	500mg single dose
Reference	39	40		41

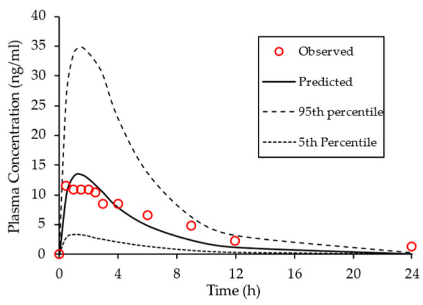

	Cmax (ng/ml)	AUC (0-24) (ng-h/ml)
Observed[a]	17.4 ± 8.7	89.0 ± 31.3
Predicted	13.9 ± 10.7	74.4 ± 58.1
Fold Error	0.80	0.84

[a]Reference 27

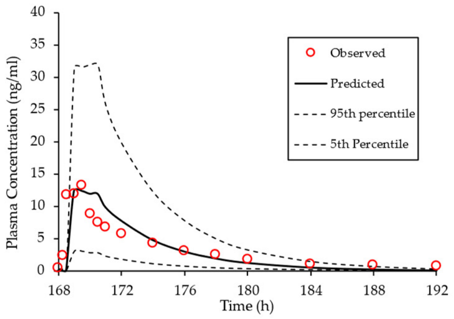

	Cmax (ng/ml)	AUC (0-24) (ng-h/ml)
Observed[a]	14.3 (12.4, 16.4[B])	62.4 (55.6, 69.2[B])
Predicted	12.7 ± 9.9	70.8 ± 53.9
Fold Error	0.89	1.13

[a]Reference 28
[B]Reported as the mean of two subjects (individual subject values in parenthesis)

Figure S1. Verification of atorvastatin compound file in Healthy Volunteer population in the Simcyp® Simulator.

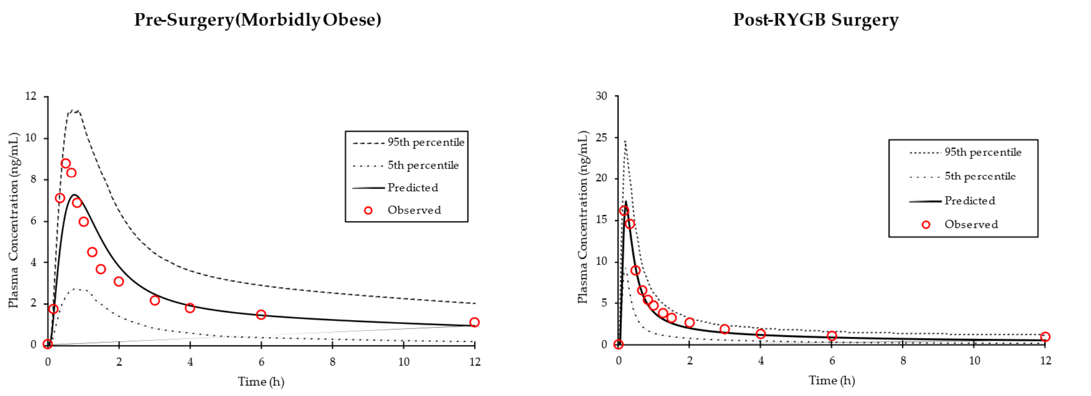

	Cmax (ng/ml)	AUC (0-24) (ng-h/ml)
Observed [a]	9.7 ± 6.7	20.8 ± 10.7
Predicted	7.4 ± 2.8	25.9 ± 12.4
Fold Error	0.76	1.25

[a]Reference 31

	Cmax (ng/ml)	AUC (0-24) (ng-h/ml)
Observed [a]	16.6 ± 7.6	18.3 ± 7.9
Predicted	17.6 ± 4.6	22.0 ± 0.35
Fold Error	1.06	1.20

[a]Reference 31

Figure S2. Verification of RYGB Surgical population using midazolam in the Simcyp® Simulator.

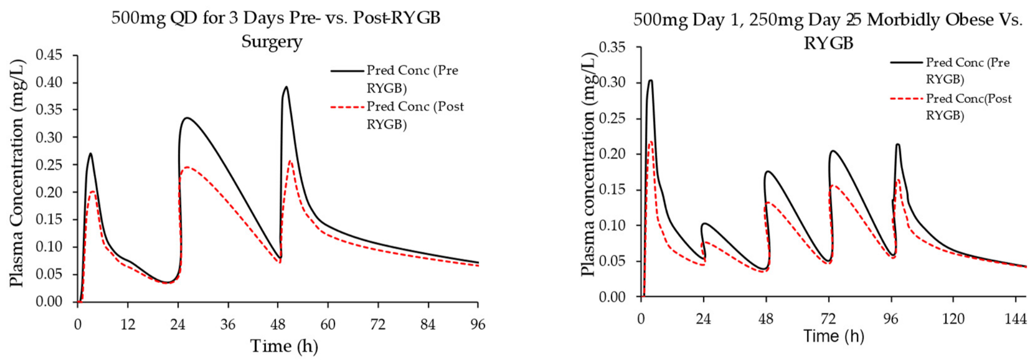

Parameter	500mg QD for 3days			500mg day 1, 250mg day 2-5		
	Pre-surgery (Morbidly Obese)	Post-surgery (RYGB Population)	Ratio	Pre-surgery (Morbidly Obese)	Post-surgery (RYGB Population)	Ratio
Cmax (mg/L)	0.39 ± 0.12	0.28 ± 0.07	0.72	0.38 ± 0.11	0.3 ± 0.06	0.74
AUC (mg.h/L)	22.5 ± 4.2	15.7 ± 5.4	0.70	20.0 ± 3.79	14.5 ± 3.4	0.72

Figure S3. PBPK model simulations of azithromycin multiple dosing regimens (tablet formulation) in pre and post-RYGB surgery populations using the Simcyp® Simulator.
